# Cannabidiol Rescues TNF-α-Inhibited Proliferation, Migration, and Osteogenic/Odontogenic Differentiation of Dental Pulp Stem Cells

**DOI:** 10.3390/biom13010118

**Published:** 2023-01-06

**Authors:** Lina Yu, Liting Zeng, Zeyu Zhang, Guanxiong Zhu, Zidan Xu, Junyi Xia, Jinlong Weng, Jiang Li, Janak Lal Pathak

**Affiliations:** 1 Guangzhou Key Laboratory of Basic and Applied Research of Oral Regenerative Medicine, Department of Preventive Dentistry, Affiliated Stomatology Hospital of Guangzhou Medical University, Guangdong Engineering Research Center of Oral Restoration and Reconstruction, Guangzhou 510182, China; 2 School and Hospital of Stomatology, Guangzhou Medical University, Guangzhou 510182, China

**Keywords:** pulpitis, dental pulp stem cells, dentin/pulp regeneration, cannabidiol, inflammation

## Abstract

Strategies to promote dental pulp stem cells (DPSCs) functions including proliferation, migration, pro-angiogenic effects, and odontogenic/osteogenic differentiation are in urgent need to restore pulpitis-damaged dentin/pulp regeneration and DPSCs-based bone tissue engineering applications. Cannabidiol (CBD), an active component of Cannabis sativa has shown anti-inflammation, chemotactic, anti-microbial, and tissue regenerative potentials. Based on these facts, this study aimed to analyze the effect of CBD on DPSCs proliferation, migration, and osteogenic/odontogenic differentiation in basal and inflammatory conditions. Highly pure DPSCs with characteristics of mesenchymal stem cells (MSCs) were successfully isolated, as indicated by the results of flowcytometry and multi-lineage (osteogenic, adipogenic, and chondrogenic) differentiation potentials. Among the concentration tested (0.1–12.5 µM), CBD (2.5 μM) showed the highest anabolic effect on the proliferation and osteogenic/odontogenic differentiation of DPSCs. Pro-angiogenic growth factor VEGF mRNA expression was robustly higher in CBD-treated DPSCs. CBD also prompted the migration of DPSCs and CBD receptor CB1 and CB2 expression in DPSCs. TNF-α inhibited the viability, migration, and osteogenic/odontogenic differentiation of DPSCs and CBD reversed these effects. CBD alleviated the TNF-α-upregulated expression of pro-inflammatory cytokines TNF-α, interleukin (IL)-1β, and IL-6 in DPSCs. In conclusion, our results indicate the possible application of CBD on DPSCs-based dentin/pulp and bone regeneration.

## 1. Introduction

Pulpitis is an inflammatory disease caused by a bacterial infection of the dental pulp tissue [1,2]. Dental pulp differs from other connective tissues since it is surrounded by dental hard tissues and communicates with the periodontal ligament predominantly through the apical foramen [3]. This low-compliance system of dental pulp is related to poor self-regulation and self-repair capacity, making dental pulp vulnerable to necrosis by trauma and infections [4,5]. The early manifestation of pulpitis is atypical, and the course of the disease is long. Failure of early treatment of pulpitis causes acute/chronic periapical periodontitis, tooth splitting, root fracture, occlusal pain, mastication difficulties, loss of tooth function, and other complications. Pulpitis-related clinical complications not only affect oral health but also increase the physiological pain of patients and reduce their quality of life [6]. In clinical practice, root canal treatment (RCT) removes the inflamed pulp and replaces it with inorganic material, which reduces inflammation, relieves pain, and results in a nonvital tooth [7,8]. However, after treatment, the tooth loses nutritional supplementation to the dental hard tissue, sensitive responsiveness to hot/cold stimulation, and the ability to defend against secondary infections [8,9,10]. At present, researchers believe that dental pulp regeneration followed by RCT revitalizes the tooth. There are studies on the use of tissue engineering technology to construct structures with dental pulp and dentin or pulp-dentin complex to play the temperature-sensitive effect of dental pulp tissue and provide nutrition supply and other functions. However, in the case of permanent teeth pulpitis without pulp necrosis, it is necessary to ensure the survival and differentiation potential of dental pulp stem cells (DPSCs). Therefore, more effective treatment methods for pulpitis need to be explored in clinical practice.

Mesenchymal stromal cells (MSCs) possess several unique properties which render them an ideal candidate for cell-based therapy. MSCs are classified according to the cell source, such as bone marrow-derived MSCs (BMSCs), adipose-derived MSCs (ADSCs), umbilical cord-derived MSCs (UC-MSCs), and umbilical cord blood-derived MSCs (UCB-MSCs) [11,12,13]. Regardless of the tissue sources, MSCs have low immunogenicity and can suppress the activation of immune cells [14]. Therefore, both autologous and allogeneic MSCs can be used clinically as therapeutic stem cells with minimal risk of rejection by the host immune system. A significant advantage of allogeneic MSCs is that the cells can be developed into an off-the-shelf product as a product for clinical application. DPSCs are derived from the ectoderm of neural crest cells and have a similar mineralization ability to bone marrow-derived mesenchymal stromal cells (BMSCs) [15,16]. In recent years, DPSCs have received extensive attention in the field of regenerative medicine. During dentin injury, DPSCs migrate, proliferate, and differentiate into odontoblasts. Differentiated odontoblasts formed reparative dentin at the injured site [17]. At the same time, studies have shown that DPSCs can not only differentiate into odontoblasts/osteoblasts, but also have the potential to differentiate into bone, cartilage, neural tissue, and blood vessels [18,19,20,21,22]. DPSCs release a variety of biological factors that are involved in cell proliferation, vascularization, cell migration, anti-apoptosis, and anti-inflammation [23,24,25,26]. These properties make DPSCs a promising source of seed cells for dentin/pulp revitalization during RCT treatment; however, the inflammatory milieu of pulpitis might undermine the DPSCs function causing failure in dentin/pulp revitalization. Various nanomaterials and growth factors such as BMPs, VEGF, and SDF-1α had been shown to promote DPSCs’ functions in vitro and animal models [27,28,29]. However, their clinical application is a question due to bio-safety concerns and possible adverse effects.

Cannabidiol (CBD) is the main cannabinoid and nonintoxicating compound extracted from Cannabis sativa plants that has gained popularity for medical uses ranging from epilepsy to pain control, anti-inflammation, and treatment of autoimmune diseases, neurodegenerative diseases, cancer, and drug addiction [30]. Because of the different mechanisms of action of CBD from marijuana, its medical application is safe and legal. A growing number of studies have found links between cannabinoids and oral health and disease [31]. Cannabinoids elicit their activity which acts on two mainly specific G protein-coupled cannabinoid receptors, CB1 and CB2 [32,33]. Recent studies have found that CB1 and CB2 receptors are expressed in dental pulp cells [34,35], and CBD can promote the migration, proliferation, and osteogenic differentiation of mesenchymal stem cells (MSCs). However, whether CBD can promote DPSCs’ functions including proliferation, migration, and osteogenic/odontogenic differentiation in pulpitis-related inflammatory milieu has not been investigated yet. Based on these facts, this study analyzed the effect of CBD on the viability/proliferation, migration, and mineralization potential of DPSCs in the absence or presence of TNF-α. Our results showed that CBD rescues the TNF-α-inhibited viability, migration, and mineralization potential of DPSCs, as well as downregulated TNF-α-induced expression of inflammatory markers in DPSCs. Our results indicate CBD as a potential therapeutic agent to treat pulpitis and DPSCs-based dentin/pulp regeneration.

## 2. Materials and Methods

### 2.1. Cell Isolation and Culture

DPSCs were collected from wisdom teeth and premolars extracted from 20 individuals aged 12 to 20 years during orthodontic treatment. This study followed the Declaration of Helsinki’s standards and was approved by the Affiliated Stomatology Hospital of Guangzhou Medical University’s ethical committee (JCYJ2022002). The recruitment was carried out between June and September 2021. All procedures were performed at the Department of Oral and Maxillofacial Surgery (School of Stomatology, Guangzhou Medical University), and written informed consent was obtained from each participant and the legal guardians of patients. The extracted teeth were placed in α-minimal essential medium (α-MEM; Gibco; Thermo Fisher Scientific, Inc.) supplemented with penicillin (100 U/mL) and streptomycin (100 mg/mL) on ice and were delivered to the laboratory. With the aid of a hammer, the extracted teeth were fractured to separate the crown from the root. The pulp tissue of the coronary portion was collected and then cut with scissors until small fragments were obtained (<0.5 mm). The pulp tissues were digested by 3 mg/mL Collagenase I (Sigma Aldrich, St. Louis, MO, USA) and 4 mg/mL Dispase II (Sigma Aldrich, St. Louis, MO, USA) at 37 ℃ for 30 min with shaking and were distributed in 35 mm Petri dishes. The DPSCs were cultured in a culture medium containing α-MEM (Gibco, Waltham, MA, USA), 15% fetal bovine serum (FBS), (Gibco, Waltham, MA, USA), and 100 U/mL penicillin/streptomycin (Gibco, Waltham, MA, USA) at 37 °C in a 5% CO_2_ incubator. The medium was refreshed every 3 days. After reaching 80% confluence, the cells were detached with 0.25% trypsin/EDTA (Gibco, Waltham, MA, USA) and passaged. DPSCs at passages 3–5 were used for experiments.

### 2.2. Identification of Surface Markers by Flow Cytometry

This experiment was carried out to determine the mesenchymal origin of the DPSCs. Cells were suspended with 0.25% trypsin and washed with phosphate-buffered saline (PBS). Along with the instruction, cells were incubated with CD34, CD45, CD44, CD73, CD90, and CD105 (BD Pharmingen and Abcam, USA) for 25 min at 25 °C. The cells were washed twice with PBS. Cells that were not treated with fluorescent antibodies were used as controls. The cell surface expression of markers was assessed and analyzed using flow cytometry (Beckman Coulter, Placentia, CA, USA).

### 2.3. Multilineage Differentiation of DPSCs

Alizarin red staining: The experiments in this section were designed to assess mineralized matrix deposits during osteogenic/odontogenic differentiation. Cells were cultured in 48-well plates and induced for 28 days in the osteogenic differentiation medium consisting of α-MEM supplemented with 10% FBS, 10 nM dexamethasone (Sigma-Aldrich, St Louis, MO, USA), 0.2 mM ascorbic acid (Sigma-Aldrich, St Louis, MO, USA), and 10 mM β-glycerophosphate disodium salt hydrate (Sigma-Aldrich, St Louis, MO, USA). The medium was refreshed every 3 days. After fixation by 4% paraformaldehyde (PFA) for 30 min, the cells were washed with deionized water and stained with alizarin red (Solarbio, Beijing, China) for 5 min at room temperature.

Oil-red O staining: This experiment was designed to determine the adipogenic differentiation potential of DPSCs. Cells were seeded in 48-well plates and grown in an adipogenic induction medium consisting of α-MEM supplemented with 10% FBS, 0.5 mM 3-isobutyl-1-methylxanthine (Solarbio, Beijing, China), 2 μM dexamethasone, 0.2 mM indomethacin (Sigma-Aldrich, St Louis, MO, USA), and 0.01 g/L insulin (Sigma-Aldrich, St Louis, MO, USA). After 28 days, the cells were stained with oil-red O (Solarbio, Beijing, China). The microscopic photographs were captured and recorded.

Alcian blue staining: Multi-lineage differentiation characteristics also include chondrogenic differentiation. DPSCs were seeded in 48-well plates and grown in a chondrogenic differentiation medium (BGsciences, Guangzhou, China) according to the manufacturer’s instructions. The medium was changed every other day. After 21 days of culture, the cells were fixed with 4% paraformaldehyde for 30 min and stained with Alcian solution (Solarbio, Beijing, China) at room temperature for 30 min. The microscopic photographs were captured and recorded.

### 2.4. Cell Counting Kit-8 (CCK-8) Assay

The CCK-8 test kit (Dojindo, Kumamoto, Japan) was used to analyze cell proliferation according to the manufacturer’s instructions. DPSCs were seeded into 96-well plates (3 × 10^3^ cells/well) and cultured with or without CBD (0, 0.1, 0.5, 2.5, and 12.5 µM) and TNF-α (20 ng/mL and 50 ng/mL). CCK8 reagent (10 µL)+ α-MEM (90 µL) were added in each well and incubated at 37 °C for 1 h and the absorbance was measured according to the manufacturer’s instructions. Each test was repeated at least three times. (Thermo Fisher Scientific, Waltham, MA, USA).

### 2.5. Alkaline Phosphatase (ALP) Staining and Activity

To assess the osteogenic phenotype of DPSCs after 4 and 7 days of culture, The cells were seeded at 1.5 × 10^4^ cells/well in 48-well plates and cultured in a complete medium for 24 h. Then, the old medium was replaced with the osteogenic/odontogenic medium with or without CBD (0, 0.1, 0.5, 2.5, and 12.5 µM) and TNF-α (20 ng/mL and 50 ng/mL). The medium was replenished every 3 days. For ALP staining on days 4 and 7, the old medium was removed and washed twice with PBS, then the cells were fixed with 4% paraformaldehyde for 30 min. The cells were washed twice with PBS and stained with the BCIP/NBT alkaline phosphatase color development kit (Beyotime, Hunan, China). The staining was observed under a bright field microscope. (Leica, Germany).

For the measurement of ALP activity, the cells were washed twice with PBS, and the lysate was extracted in a lysis buffer containing 0.1% Triton x-100. The total protein concentration was determined using the BCA protein assay reagent kit (Beyotime, Hunan, China). The activity of ALP was determined using an alkaline phosphatase assay kit (Beyotime, Hunan, China) and the absorbance was read at 405 nm with a microplate reader (Synergy) after incubation at 37 °C for 20 min. The value of ALP activity was normalized.

### 2.6. Alizarin Red S (ARS) Staining

Cells were seeded at 1.5 × 10^4^ cells/well in 48-well plates and cultured in a complete medium for 24 h. Then, the old medium was replaced with the osteogenic/odontogenic medium with or without CBD (0, 0.1, 0.5, and 2.5 µM) and TNF-α (50 ng/mL), respectively. The medium was refreshed every 3 days. On day 14, after removing the old medium and washing it twice with PBS, the cells were fixed with 4% paraformaldehyde for 30 min. The cells were washed twice with PBS and stained by ARS staining (Solarbio, Beijing, China) for 5 min at room temperature. The microscope was used for observation and photography. For semiquantitative analysis, OD values of the solution at 562 nm wavelength were recorded after the mineralized nodules were completely eluted by 10% cetylpyridinium chloride (CPC) solution (Sigma-Aldrich, St Louis, MO, USA) for 30 min.

### 2.7. RNA Isolation and Real-Time Quantitative PCR (RT-qPCR) Analysis

The total RNA from DPSCs was extracted by a total RNA extraction kit (Accurate Biotechnology, Hunan, China). cDNA synthesis was performed using 500 ng total RNA in a 10 µL reaction mix consisting of 2 uL transcriptor reverse transcriptases (Accurate Biotechnology, Hunan, China). RT-qPCR was performed using an SYBR Green RT-qPCR kit (Accurate Biotechnology, Hunan, China). Relative mRNA expression was normalized to that of the internal GAPDH control. The primer sequences are listed in Table 1. The relative expression of targeted genes was calculated by the 2^−ΔΔCt^ method. Each test was repeated at least three times.

### 2.8. Western Blot Assay

The total protein was extracted using RIPA buffer (Beyotime, Nanjing, China) with protease inhibitor (PMSF, Beyotime, Nanjing, China) on ice for 30 min and collected the supernatant was by centrifuging at 13,450 rpm for 15 min. The total protein concentration was measured using the BCA protein assay kit (Beyotime, Nanjing, China). Total protein extracts (20 µg) from cells were separated via 10% SDS-PAGE (Epizyme, Shanghai, China), transferred to 0.45 mm polyvinylidene fluoride (PVDF) membranes (Millipore, Burlington, MA, USA), blocked with 5% non-fat milk for 1 h at room temperature, and washed three times with TBST (5 min each time). The membrane was incubated with the primary antibody overnight at 4 °C, which included rabbit anti-human Collagen I (COL-I, Proteintech, Wuhan, China), and rabbit anti-human RUNX2 (Proteintech, Wuhan, China). On the second day, the primary antibodies were removed and the membrane was cultured with the second antibody for 1 h at room temperature. After incubation with the primary antibody and the secondary antibody, the target protein was visualized by chemiluminescence using an enhanced chemical luminescence kit (Beyotime, Nanjing, China). The ImageJ software was used for the semi-quantification of band intensity.

### 2.9. Scratch Wound Healing Assay

DPSCs were cultured in 6-well plates for 12 h before scratching. A 200 µL pipette tip was firmly pressed against the top of the tissue culture plate to swiftly create a vertical wound through the cell monolayer and then washed with serum-free medium to remove detached cells. The cells were incubated with an FBS-free culture medium with or without CBD (2.5 μM) and/or TNF-α (20 ng/mL and 50 ng/mL) for 24 and 48 h, the wound area was captured using microscopy (Leica, Wetzlar, Germany) and Image J software to quantify the wound area.

### 2.10. Immunofluorescence Staining

After treatment without or with CBD (2.5 μM) and TNF-α (50 ng/mL) for 3 days, DPSCs were fixed with 4% paraformaldehyde solution at 37 °C for 20 min, treated with QuickBlock™ Blocking Buffer (Beyotime, China) for 10 min. Expression of osteogenic/odontogenic markers was analyzed by immunofluorescence staining using primary antibodies Osteopontin (OPN, 1:100, Proteintech, Wuhan, China) and primary antibodies COL-I (1:100, Proteintech, Wuhan, China) at 4 °C overnight, followed by incubation with fluorophore-conjugated secondary antibodies (1:400, Proteintech, Wuhan, China) for 1 h at room temperature. DAPI staining for 10 min was carried out after secondary antibody incubation. The cytoskeleton was stained with Phalloidine. Staining was detected using fluorescent microscopy (Olympus, Tokyo, Japan). Image J software was used for quantification.

### 2.11. Statistical Analysis

Data are presented as mean ± standard deviation (SD). Data were analyzed using Graphpad Prism 8.0 (GraphPad Software Inc., La Jolla, CA, USA). One-way analysis of variance (ANOVA) or Student’s t-test was used to test differences between groups. A *p*-value < 0.05 was considered statistically significant.

## 3. Results

### 3.1. Isolation and Characterization of DPSCs

**Figure 1:**
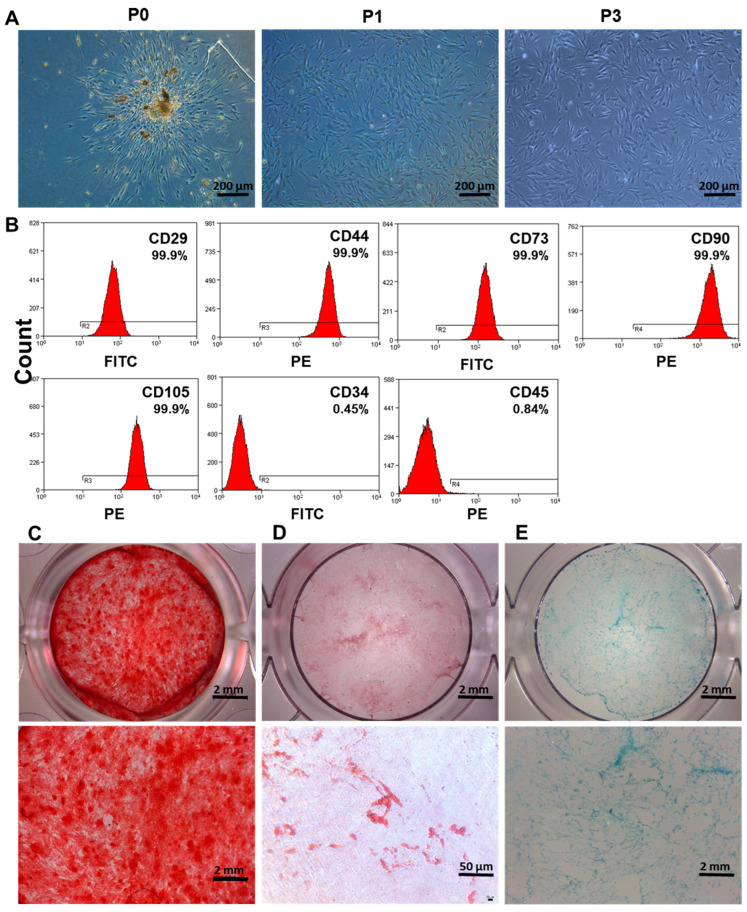
Isolation and identification of DPSCs. (**A**) Microscopic images of human DPSCs culture at primary passage (P0), passage 1 (P1), and passage 3 (P3). (**B**) Analysis of DPSCs surface markers by flow cytometry. (**C**) Alizarin red, (**D**) oil red O, and (**E**) alcian blue staining of DPSCs after inducing osteogenic, adipogenic, and chondrogenic differentiation, respectively.

The DPSCs have a spindle-like form and are placed in a whirlpool pattern (Figure 1A). The immunophenotype of cells was analyzed by flow cytometry CD44, CD73, CD90, and CD105 were positive for mesenchymal stem cell markers, whereas CD34 and CD45 were negative (Figure 1B). DPSCs showed multilineage differentiation potential. After four weeks of osteogenic induction, the mineralized nodules were observed by alizarin red staining (Figure 1C). Under adipogenic induction, red lipid droplets were detected at 28 days of culture (Figure 1D). Similarly, under chondrogenic induction for 28 days, DPSCs culture was positive for Alcian blue staining (Figure 1E). Our results indicate mesenchymal stem cell phenotype of DPSCs.

### 3.2. CBD-Induced Proliferation and Osteogenic/Odontogenic Differentiation of DPSCs

**Figure 2:**
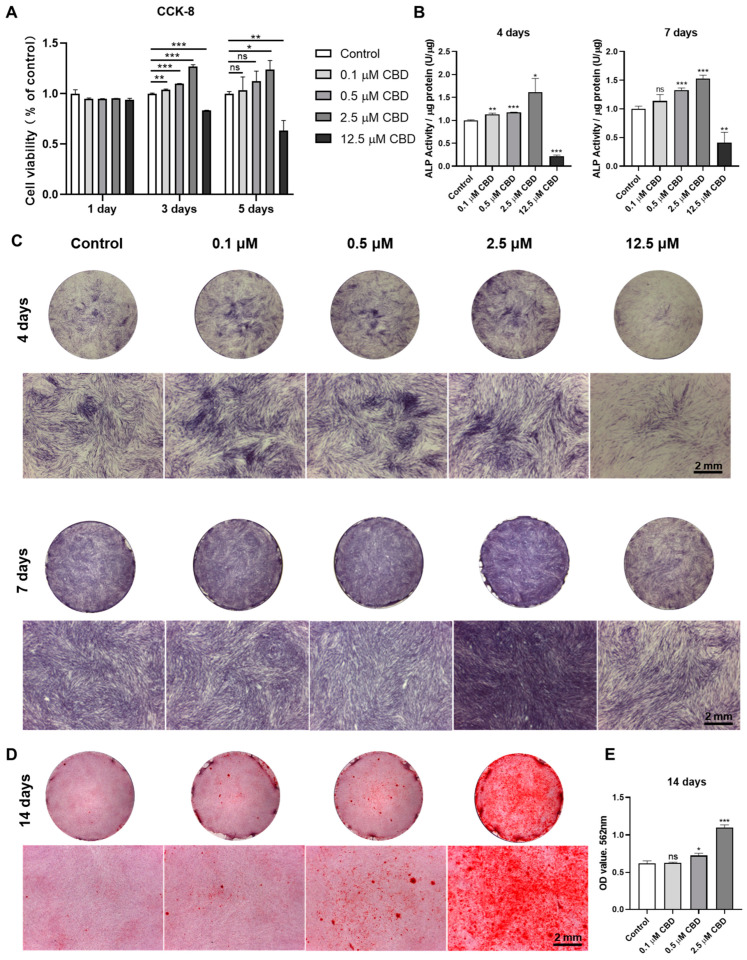
CBD promotes the cell viability and odontogenic/osteogenic differentiation ability of hDPSCs. (**A**) The cell viability of DPSCs was detected by CCK-8 assay. (**B**) Alkaline phosphatase activity. (**C**) Alkaline phosphatase staining. (**D**) Alizarin red staining. (**E**) Quantification of the mineralized matrix from alizarin red staining. Scale bar: 2 mm. Significant effect of the treatment compared to the control group, * *p* < 0.05, ** *p* < 0.01, and *** *p* < 0.001.

To investigate the effect of CBD on DPSCs proliferation, cells were treated with CBD at different concentrations (CBD: 0, 0.1, 0.5, 2.5, and 12.5 µmol/L) (Figure 2A). CBD at concentrations below 2.5 μM enhanced DPSCs proliferation at day 3 and 5 of culture. However, CBD at 12.5 μM concentration dramatically inhibited cell proliferation at day 3 and 5 of culture, indicating the cytotoxic effect of this high dose of CBD on DPSCs.

CBD promoted the ALP staining of DPSCs at 0.1–2.5 μM concentrations (Figure 2C). Similar to the ALP staining results, analysis of ALP activity demonstrated that CBD promoted osteogenic/odontogenic differentiation of DPSCs, especially at 2.5 μM concentration (Figure 2B). CBD at a concentration of 12.5 μM dramatically inhibited ALP production and activity of DPSCs. After 14 days of osteogenic/odontogenic induction, alizarin red staining showed increased matrix mineralization in CBD-induced group. Notably, 2.5 μM CBD significantly increased matrix mineralization at 14 days of culture (Figure 2D,E). Among the concentration tested, 2.5 μM CBD exerted the highest effect on the proliferation and osteogenic differentiation of DPSCs.

### 3.3. CBD Upregulated the Expression of Osteogenic/Odontogenic and Angiogenic Markers in DPSCs

**Figure 3:**
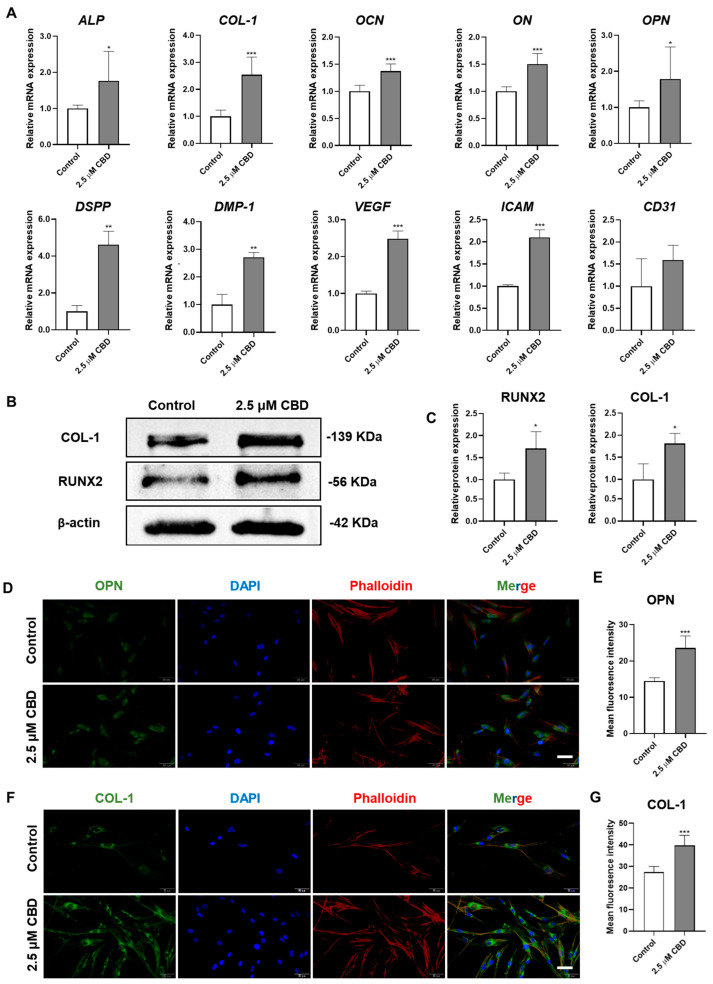
CBD upregulates the expression of osteogenic/odontogenic and angiogenic markers in DPSCs. (**A**) mRNA level expression pattern of osteogenic/odontogenic and angiogenic markers in DPSCs. (**B**,**C**) Western blot analysis and quantification of COL−I and RUNX2 at day 14. Immunohistochemistry analysis of OPN (**D**,**E**) and COL−I (**F**,**G**) expression in DPSCs at day 3. Scale bar: 50 μm. Significant effect of the treatment compared to the control group, * *p* < 0.05, ** *p* < 0.01, and *** *p* < 0.001.

CBD robustly upregulated the mRNA level expression of osteogenic/odontogenic differentiation markers alkaline phosphatase (ALP), osteopontin (OPN), osteocalcin (OCN), osteonectin (ON), collagen type I (COL−I), dentin sialophosphoprotein (DSPP), and dentin matrix protein-1 (DMP−1) in DPSCs (Figure 3A). Similarly, CBD also upregulated the mRNA level expression of angiogenic markers VEGF and ICAM in DPSCs. The results of Western blot analysis also confirmed the osteogenic/odontogenic differentiation-inducing potential of CBD as indicated by the upregulation of RUNX2 and COL-1 proteins in DPSCs (Figure 3B,C). Immunofluorescence staining also showed higher expressions of OPN and COL-1 in DPSCs cultured with CBD (Figure 3D,E).

### 3.4. CBD Promoted DPSCs Migration and Upregulated the Expression of CBD Receptors CB1 and CB2 in DPSCs

**Figure 4:**
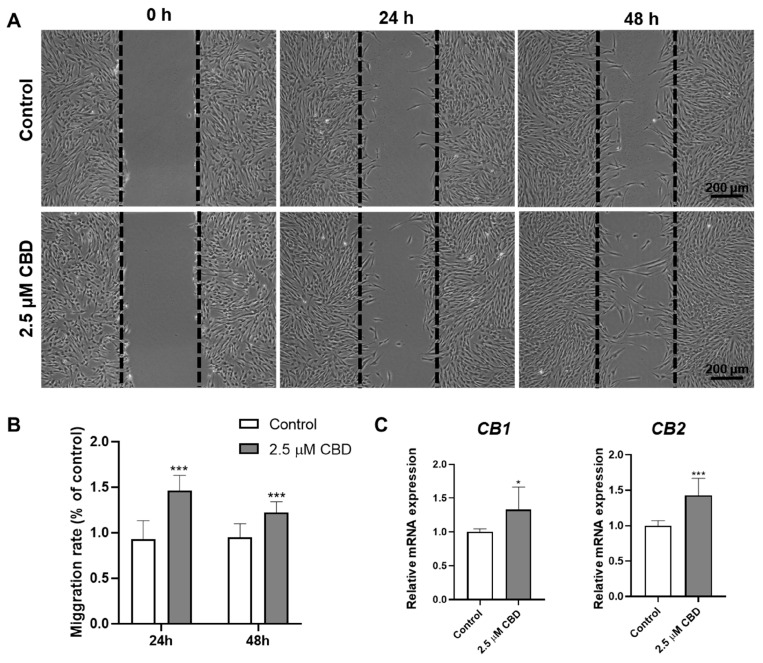
CBD promotes DPSCs’ migration and upregulates the expression of CBD receptors CB1 and CB2 in DPSCs. (**A**) Representative images of scratch wound closure assay. (**B**) Quantification of DPSCs’ migration rate, n = 5. (**C**) mRNA level expression pattern of CB1 and CB2. Significant effect of the treatment compared to the control group, * *p* < 0.05 and *** *p* < 0.001.

CBD (2.5 μM) promoted the migration of DPSCs in scratch wound healing assay at both 24 and 48 h of culture (Figure 4A,B). Similarly, we analyzed the expression of CB1 and CB2 receptors in DPSCs after CBD treatment and found that both the CBD receptors were upregulated in DPSCs after CBD treatment (Figure 4C).

### 3.5. CBD Alleviated the TNF-α-Mediated Inhibitory Effect in DPSCs Viability, Migration, and Osteogenic/Odontogenic Differentiation

**Figure 5:**
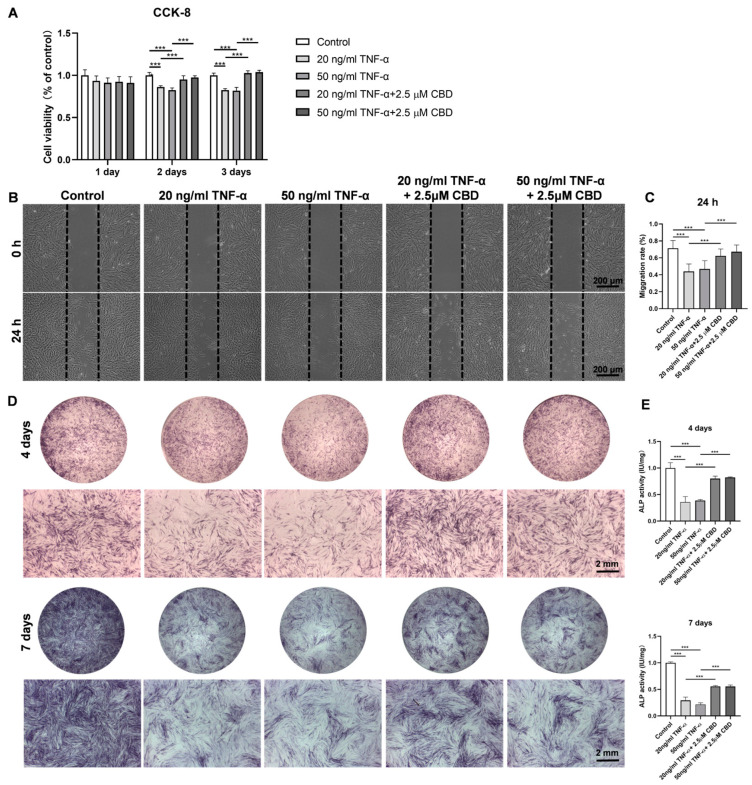
CBD abrogates the negative effect of TNF-α on DPSCs’ viability, migration, and osteogenic/odontogenic differentiation. (**A**) The effect of different concentrations of TNF-α and TNF-α + CBD on the cell viability of DPSCs. (**B**) Representative images of scratch wound closure assay showing DPSCs’ migration. (**C**) The average area migrated into the gap from time 0 to the indicated time points was analyzed and measured using Image J software. (**D**) Alkaline phosphatase staining at day 4 and 7. (**E**) Alkaline phosphatase activity at day 4 and 7. Significant effect of the treatment compared to the control group, *** *p* < 0.001.

**Figure 6:**
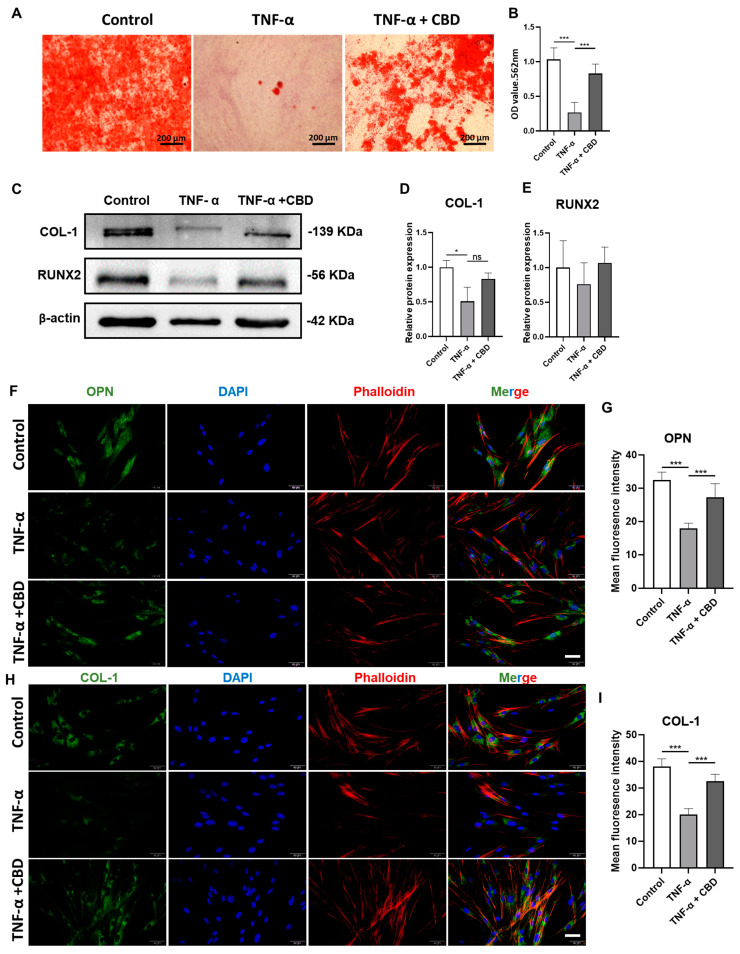
CBD rescues TNF-α-inhibited osteogenic/odontogenic differentiation of DPSCs. (**A**) Alizarin red staining of DPSCs culture at day 28. (**B**) Quantification of the mineralized matrix from alizarin red staining. Scale bar: 2 mm. (**C**) Western blot analysis for COL−I and RUNX2 at day 7. Quantification of COL−I (**D**) and RUNX2 (**E**) expression from Western blots. Immunohistochemistry analysis quantification of OPN (**F**,**G**) and COL−I (**H,I**) expression in DPSCs. Scale bar: 50 μm. Significant effect of the treatment compared to the control group, * *p* < 0.05, *** *p* < 0.001.

TNF-α (20 ng/mL and 50 ng/mL) inhibited the viability of DPSCs at day 2 and 3 of culture (Figure 5A). This inhibitory effect of TNF-α on DPSCs viability was reversed by CBD (2.5 μM) treatment. Similarly, TNF-α (20 ng/mL and 50 ng/mL) inhibited DPSCs migration (Figure 5B,C). CBD (2.5 μM) treatment also rescued the TNF-α-mediated inhibition of DPSCs migration. We also investigated the effect of TNF-α on the osteogenic/odontogenic differentiation of DPSCs. TNF-α (20 ng/mL and 50 ng/mL) inhibited ALP production and activity on both day 4 and 7 (Figure 5D,E). CBD (2.5 μM) rescued the inhibitory effect of TNF-α on ALP production and activity in DPSCS. TNF-α (50 ng/mL) dramatically inhibited the matrix mineralization by DPSCs at day 28, and this effect was reversed by the CBD (2.5 μM) treatment (Figure 6A,B). Western blot analysis also revealed the inhibitory effect of TNF-α (50 ng/mL) on COL-1 expression and CBD (2.5 μM) treatment reversed this effect (Figure 6 A,B). Although decreased trend of RUNX2 expression in the TNF-α (50 ng/mL) group and an increasing trend in the TNF-α + CBD treated group was observed, the difference was not statistically significant (Figure 6C,E). Immunofluorescence staining revealed the downregulation of OPN and COL-1 in TNF-α (50 ng/mL) treated DPSCs, and this effect was reversed after CBD treatment (Figure 6F–I).

### 3.6. CBD Attenuated TNF-α-Induced Expression of Inflammatory Markers in DPSCs

**Figure 7:**
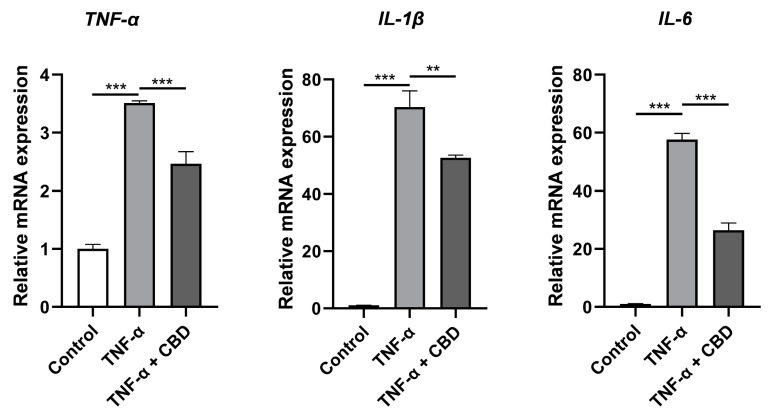
CBD alleviates TNF-α upregulated expression of inflammatory cytokines in DPSCs. Significant effect of the treatment compared to the control group, ** *p* < 0.01 and *** *p* < 0.001.

To assess cytokine secretion in response to CBD (2.5μM) and TNF-α (50 ng/mL), DPSCs were treated with either TNF-α alone or with both TNF α and CBD for 24 h. Data from RT-qPCR showed that the mRNA expression of TNF α, IL 6, and IL 1β in the TNF-α-treated group was significantly increased compared with that in the control group. Interestingly, CBD treatment attenuated the TNF-α-induced expressions of TNF-α, IL 6, and IL 1β in DPSCs (Figure 7). These results indicated that CBD attenuates the inflammatory effects of TNF-α in DPSCs.

## 4. Discussion

**Figure 8:**
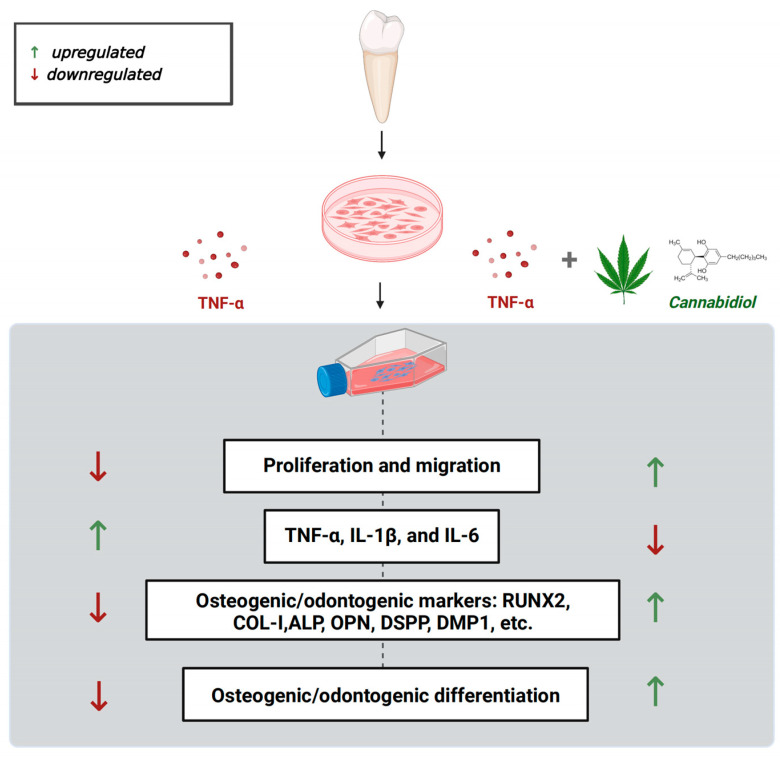
Scheme showing the protective effect of CBD against TNF-α mediated catabolic effects on DPSCs’ functions.

An ideal approach for restoring pulp structure and function is to promote the stemness, migration, osteogenic/odontogenic, and pro-angiogenic ability of DPSCs. DPSCs also have shown bone regenerative potential. Reports from the literature have shown the anti-inflammatory, antioxidant, antibacterial, and hard/soft tissue regenerative potential of CBD [35,36,37]. However, maintaining the DPSCs’ vitality and functions in the inflammatory condition is still a challenge that hinders the application of DPSCs in dentin/pulp and bone regeneration applications. In this study, we found that CBD promotes the viability, migration, and osteogenic/odontogenic differentiation potential of DPSCs. Moreover, CBD treatment upregulated the mRNA expression of pro-angiogenic markers in DPSCs. CBD rescued the TNF-α-inhibited viability, migration, and osteogenic/odontogenic differentiation of DPSCs. CBD treatment also inhibited the TNF-α-induced mRNA expression of pro-inflammatory markers in DPSCs. These results indicate CBD as a potential therapeutic agent for DPSCs-based dentin/pulp revitalization and bone regeneration in basal and inflammatory conditions (Figure 8).

DPSCs, a type of MSCs, have robust proliferation and multi-lineage differentiation potential [38]. DPSCs isolated in this study showed higher expression (99.9%) of MSCs markers CD29, CD44, CD73, CD90, and CD105. Moreover, DPSCs showed robust osteogenic, adipogenic, and chondrogenic differentiation potential. These findings of our study are in accordance with the reports from the literature [39]. Since DPSCs are the key cell population in the dental pulp, pulpitis-related inflammation could directly affect the DPSCs survival and functions including odontogenesis, pro-angiogenesis, immunomodulation, etc. [40] Similarly, DPSCs have frequently used seed cells for dentin/pulp revitalization [35,41]. Various drugs and growth factors including BMP2, lithium chloride, etc. had been tested for their potential to promote DPSCs functions related to dental pulp revitalization [28,42]. In this study, we tested the efficacy of CBD in promoting DPSCs function in physiological conditions as well as in pulpitis-related inflammatory conditions. CBD at 0.1 to 2.5 µM promoted DPSCs’ proliferation but 12.5 µM inhibited DPSCs’ proliferation. Among the concentration tested, 2.5 µM CBD showed the highest anabolic effect on DPSCs’ proliferation and osteogenic/odontogenic differentiation. Reports from the literature had shown 5 µM CBD promotes osteogenic/odontogenic differentiation of human dental pulp cells [35]. Our reports also showed that CBD promoted the proliferation, migration, odontogenesis, and osteogenic differentiation of DPSCs, but 2.5 μM CBD is the optimal concentration. Although optimal concentrations are inconsistent, low concentrations (<10 μM) of CBD seem to promote the proliferation, migration, and pro-angiogenic and osteogenic/odontogenic potential of DPSCs. At the same time, Schönhofen P et al. reported that exposure to 2.5 µM CBD during neuronal differentiation could sensitize immature cells to future challenges with neurotoxins [43]. Results of our study and reports from the literature indicate 2.5 µM CBD as the optimal concentration to promote the viability and osteogenic/odontogenic differentiation of DPSCs.

Angiogenesis plays a crucial role in dentin/pulp regeneration and pulp revitalization as well as bone regeneration [44]. DPSCs express a plethora of angiogenic factors to induce angiogenesis directly or via endocrine effects [45,46,47]. Among these factors, VEGF is a key angiogenic factor overexpressed by DPSCs [48]. We found the robust overexpression of VEGF in CBD-treated DPSCs. This result indicates that CBD promotes the pro-angiogenic potential of DPSCs. However, further angiogenesis experiments in CBD-pretreated DPSCs and endothelial cell co-culture are required to confirm this result. DPSCs migration plays a vital role in dentin and pulp regeneration. CBD promoted the migration of DPSCs and the expression of CB1 and CB2 receptors in DPSCs. Schmuhl et al. reported similar effects of CBD in migration and CB receptors expression of adipose tissue-derived MSCs [49]. Our results indicate a possible stimulatory effect of CBD on proangiogenic potential and migration of DPSCs.

Pulpitis-induced pro-inflammatory milieu in the dental pulp plays a vital role in dentin degeneration and pulp necrosis [50]. Therefore, mitigation of inflammation and/or the catabolic effect of inflammation in DPSCs are crucial for pulpitis treatment and revitalization of pulpitis-damaged dentin and pulp. Similarly, the inflammatory milieu in the periodontal region hinders stem cell-based alveolar bone regeneration. Levels of various pro-inflammatory cytokines including TNF-α are upregulated in the pulpitis-affected pulp tissue and periodontitis-affected alveolar bone [51,52]. TNF-α is frequently used in vitro cell cultures as an inflammatory cytokine mimicking the inflammatory situation in vivo [53]. TNF-α inhibited the cellular activities of DPSCs including viability, migration, and osteogenic/odontogenic differentiation. Interestingly, CBD treatment nullified these catabolic effects of TNF-α in DPSCs function. This is the first study to report the potential of CBD on rescuing the inflammation-induced catabolic effects in DPSCs’ functions. Reports from the literature have shown the anti-inflammatory effect of CBD in neuron and intestinal tissues [54,55]. CBD inhibits LPS-induced inflammation in macrophages, lung epithelial cells, and fibroblasts [56]. DPSCs modulate inflammation during pulpitis by overexpressing pro-inflammatory cytokines [57]. We found that TNF-α treatment robustly promoted the expression of proinflammatory markers TNF-α, IL-1β, and IL-6 in DPSCs. Interestingly, CBD treatment alleviated the TNF-α-induced expression of these pro-inflammatory cytokines in DPSCs. These results indicate the anti-inflammatory effect of CBD on DPSCs.

## 5. Conclusions

We tested the effect of CBD on DPSCs functions required for dentin and pulp revitalization and bone regeneration, including viability, migration, osteogenic/odontogenic differentiation, pro-angiogenic potential, and anti-inflammatory effects in vitro experiments. Our results showed the anabolic effect of CBD in these functions of DPSCs both in the basal and inflammatory situations suggesting the possible application of CBD or/and DPSCs on oral tissue regeneration including dentin/pulp and bone. Our results warrant in situ studies using dentin/pulp and bone regeneration models to further confirms these anabolic roles of CBD.

## Figures and Tables

**Table 1:** Primer sequences used in this study.

Gene	Acc. No	Primer Sequence (5′–3′)	Product Length (bp)
ALP	NM_001127501.4	F: AACATCAGGGACATTGACGTG R: GTATCTCGGTTTGAAGCTCTTCC	159
OPN	NM_001040060.2	F: GAAGTTTCGCAGACCTGACAT R: GTATGCACCATTCAACTCCTCG	91
OCN	NM_199173.6	F: CACTCCTCGCCCTATTGGC R: CCCTCCTGCTTGGACACAAAG	112
COL-I	NM_000088.4	F: GAGGGCCAAGACGAAGACATC R: CAGATCACGTCATCGCACAAC	140
TNF-α	NM_000594.4	F: GAGGCCAAGCCCTGGTATG R: CGGGCCGATTGATCTCAGC	91
IL-1β	NM_000576.3	F: ATGATGGCTTATTACAGTGGCAA R: GTCGGAGATTCGTAGCTGGA	132
IL-6	NM_001371096.1	F: ACTCACCTCTTCAGAACGAATTG R: CCATCTTTGGAAGGTTCAGGTTG	149
DSPP	NM_014208.3	F: TGGCGATGCAGGTCACAAT R: CCATTCCCACTAGGACTCCCA	249
DMP1	NM_001079911.3	F: CACTCAAGATTCAGGTGGCAG R: TCTGAGATGCGAGACTTCCTAAA	75
ICAM	NM_000201.3	F: ATGCCCAGACATCTGTGTCC R: GGGGTCTCTATGCCCAACAA	112
VEGF	NM_001025366.3	F: GGAGGCAGAGAAAAGAGAAAGTGT R: TAAGAGAGCAAGAGAGAGCAAAAGA	175
CD31	NM_000442.5	F: CTCCAGACTCCACCACCTTAC R: GAACTTTGCCTATTTCTTACCA	243
GAPDH	NM_001357943.2	F: GAAGGTGAAGGTCGGAGTCA R: GAAGATGGTGATGGGATTTC	172
ON CB1 CB2	NM_001309444.2 NM_001365874.3 XM_047444833.1	F:TCTTCCCTGTACACTGGCAGTTC R:AGCTCGGTGTGGGAGAGGTA F:GTGTTCCACCGCAAAGATAGC R:GGGGCCTGTGAATGGATATGT F:AGCCCTCATACCTGTTCATTGG R:GTGAAGGTCATAGTCACGCTG	73 130 154

## Data Availability

All relevant data of this study are presented. Additional data will be provided upon request.
